# Exploring Sampling in the Detection of Multicategory EEG Signals

**DOI:** 10.1155/2015/576437

**Published:** 2015-04-21

**Authors:** Siuly Siuly, Enamul Kabir, Hua Wang, Yanchun Zhang

**Affiliations:** ^1^Centre for Applied Informatics, College of Engineering and Science, Victoria University, P.O. Box 14428, Melbourne, VIC 8001, Australia; ^2^School of Agricultural, Computational and Environmental Sciences, University of Southern Queensland, Toowoomba, QLD 4350, Australia; ^3^School of Computer Science, Fudan University, Shanghai 200433, China

## Abstract

The paper presents a structure based on samplings and machine leaning techniques for the detection of multicategory EEG signals where random sampling (RS) and optimal allocation sampling (OS) are explored. In the proposed framework, before using the RS and OS scheme, the entire EEG signals of each class are partitioned into several groups based on a particular time period. The RS and OS schemes are used in order to have representative observations from each group of each category of EEG data. Then all of the selected samples by the RS from the groups of each category are combined in a one set named RS set. In the similar way, for the OS scheme, an OS set is obtained. Then eleven statistical features are extracted from the RS and OS set, separately. Finally this study employs three well-known classifiers: *k*-nearest neighbor (*k*-NN), multinomial logistic regression with a ridge estimator (MLR), and support vector machine (SVM) to evaluate the performance for the RS and OS feature set. The experimental outcomes demonstrate that the RS scheme well represents the EEG signals and the *k*-NN with the RS is the optimum choice for detection of multicategory EEG signals.

## 1. Introduction

Efficiently detecting multicategory EEG signals is beneficial for handling neurological abnormalities and also for evaluating the physiological state of the brain for a broad range of applications in biomedical community. EEG signals indicate the electrical activity of the brain and contain useful information about the brain state to study brain function [1]. The identification of different categories EEG signals is traditionally performed by experts based on the visual interpretation. The manual scoring is subject to human errors and it is time consuming, costly process and not sufficient enough for reliable information [2, 3]. Thus there is an ever-increasing need for developing automatic systems to evaluate and diagnose multicategory EEG signals to prevent the possibility of the analyst missing information. Complex characteristics of EEG signals (e.g., poor signal-to-noise ratio, nonstationary, and aperiodic) require employment of robust detection algorithms in order to achieve reasonable detection performance. Hence, designing efficient detection algorithms has been an important goal and highly attractive area to ensure a proper evaluation and treatment of neurological diseases for this study.

In order to perform the detection of signal's category, first the most important task is to extract distinguishing features or characteristics from EEG data that can describe the morphologies or the key properties of the signals. The features significantly affect the accuracy of detecting EEG signals [4]. The features characterizing the original EEG are used as the input of a classifier to differentiate different categories of EEGs. As optimal features play a very important role in the performance of a classifier, this study intends to find out a robust feature extraction process for the detection of multicategory EEG signals.

Recently, various approaches for automatic detection of multicategory EEG signals have been reported. Siuly and Li [5] proposed a statistical framework for multiclass EEG signal classifications. They developed an optimum allocation scheme based on the variability of observation within a group (based on specific time) of the EEG data and selected a representative sample. The representatives were fed to the least square support vector machine (LS-SVM) classifier instead of taking representative features that may be a limit for further consideration of a detection technique. An approach based on a cascade of wavelet-approximate entropy was introduced by Shen et al. [6] for the feature extraction in the EEG signal classification. They tested three existing methods, support vector machine (SVM), *k*-nearest neighbour (*k-*NN), and radial basis function neural network (RBFNN), and determined the classifier of best performance. Acharjee and Shahnaz [7] had a study on twelve Cohen class kernel functions to transform EEG data in order to facilitate the time frequency analysis. The transformed data formulated a feature vector consisting of modular energy and modular entropy, and the feature vector was fed to an artificial neural network (ANN) classifier. Muthanantha Murugavel et al. [8] had conducted a study based on Lyapunov feature and a multiclass SVM for the detection of EEG signals. Übeyli [9] presented an approach that integrated automatic diagnostic systems with spectral analysis techniques for EEG signal classification. The wavelet coefficients and power spectral density (PSD) values obtained by eigenvector methods were used as features, and these features were fed to each of the seven classification algorithms (SVM, recurrent neural networks (RNN), PNN, mixture of experts (ME), modified mixture of experts (MME), combined neural networks (CNN), and multilayer perceptron neural network (MLPNN)). Übeyli [10] provided another algorithm based on eigenvector methods and multiclass SVMs with the ECOC for the classification of EEG signals. In the feature extraction stage, three eigenvector methods such as the Pisarenko, MUSIC, and minimum norm were used to obtain the PSD values of the EEG signals that were employed as the input of the multiclass SVMs. For the detection of multiclass EEG signals, Guler and Ubeyli [11] had examined again SVM, PNN and MLPNN on wavelet coefficients and lyapunov exponents features. The experimental outcomes of that research demonstrated that the SVM classifier performed better than the other two classifiers with these features.

In the literatures, the majority of the existing methods cannot appropriately handle a large amount of EEG data due to their structure. On the other hand, most of the methods were limited in their success and effectiveness [10, 11]. In addition, some of the existing methods of the feature extraction stage are not the right choice for getting representative features from the original EEG data due to its nonstationary and aperiodic characteristic (e.g., Fourier transformation) [12]. Although numerous methods have been developed for feature extraction stage, little attention has been paid in the using of sampling, which is a fundamental component in statistics to represent information from original entire EEG signals. Sampling is very effective if the population (a group of observations) is heterogeneous and is very large in size. An effective sample (a subset of the group of observations) of a population represents an appropriate extraction of the useful data which provides meaningful knowledge of the important aspects of the population. It will be more expedient if the population is divided into several groups according to a specific characteristic and then selects representative samples from each and every subgroup depending on group size such that the entire samples reflect the whole population. As EEG recordings normally include a vast amount of data and the data is generally heterogeneous with respect to time period, it is a natural expectation that dividing the whole EEG recordings into some subgroups with respect to time and then taking representative samples from each subgroup would improve the performance of a classifier. This improvement is achieved in this paper for classifying the multicategory EEG signals.

Challenging these issues, this paper explores the idea of the sampling for getting representative information out from raw EEG data for the detection of multicategory EEG signals. In this study, we develop a structure for the detection of multicategory based on sampling for the feature extraction stage proposing two schemes: random sampling (RS), optimum allocation sampling (OS). This study uses the RS and OS schemes to evaluate how efficient they are to select representative samples from each segment of each category of EEG data discussed in detail in Section 2.1. “Representative sample” means a sample that is selected randomly from a segment (a short time window) called “population” and each observation of the population has a known, nonzero chance of being selected in the sample. In the proposed approach, firstly we segment the whole EEG signals of a class (a category) into several groups according to a particular time period. Then we draw samples from each group of a class using the RS and OS technique, separately. After that, for each of the RS and OS schemes, we make two separate sample sets called “RS” set and “OS” set combining all of their samples from each group of that class (detailed discussion in Section 2.1). After that we extract descriptive features from the RS set and the OS set of that class (discussed in Section 2.2). The same procedure applies for all of the classes of EEG data. The accumulation of all features from all of the classes constitutes a feature vector for the RS scheme and also for the OS scheme. The collection of all features from all class of EEG signals for the RS and OS scheme is employed as an input set in the classifier.

To achieve a higher detection performance, the set of input features and the choice of the machine learning techniques are crucial. If a feature provides large interclass differences for different classes, the technique can exhibit a better performance. In order to find out an effective model with highest accuracy for detection of multicategory EEG signals, in this paper we test three machine learning techniques, namely, *k*-nearest neighbours (*k*-NN), multinomial logistic regression with a ridge estimator (MLR), and support vector machine (SVM) on the composite features. To evaluate the performance of the classifiers, we apply cross validation procedure to create training set and testing set. All possible performance parameters are used to assess the effectiveness of the proposed approaches. It is important to note that the sample selection procedure in both the RS and OS schemes are repeated for 20 times with the reported three classifiers to observe the consistency of the structure. We also compare our proposed algorithms with the other existing well-known algorithms in the literature. The experimental results show that the proposed RS based algorithm can detect perfectly for each class of EEG signals in terms of all possible detection parameters by using the *k*-NN classifier.

The rest of the paper is organized as follows.  Section 2 presents a description of the proposed methodology in detail. In this section, we also briefly describe the three classifiers and the features extraction methods used in this paper. The description of benchmark EEG data and experimental design are provided in Section 3. In Section 4, we present the experimental results of the three classifiers with a detailed discussion. This section also provides a comparative report in the context of existing studies in the literature. Finally, concluding remarks are included in Section 5.

## 2. Method

**Figure 1:**
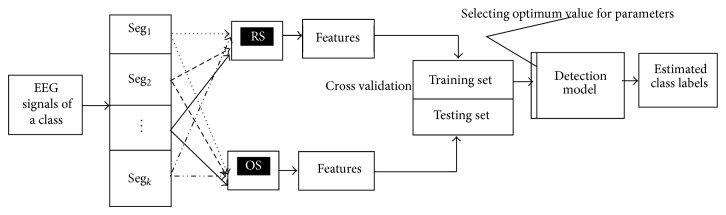
Structure of the proposed method for the detection of multicategory EEG signals.

The detection technique that is developed in this study is comprised of three key structures.* The first one* is to select representative samples from each and every segment of an entire signal data of a category (e.g., healthy subject with eye open; epileptic patient during seizure activity). In order to select a representative sample, we employ random sampling (RS) and optimum allocation sampling (OS) scheme, separately to compare their effectiveness. Then we select samples by using the RS and OS techniques from each segment of a class and consequently make two different groups (“RS” and “OS”) as shown in Figure 1. The subsequent* second one* is to extract representative features from each of the RS and OS groups to represent the distribution of data pattern and then to integrate all of the features of each class in a matrix that is called feature vector set.* The third one* is the use of detection method, which is based on the machine learning algorithms. We herewith employ three different classifiers: *k*-NN, MLR, and SVM for the detection of multicategory EEG signals. Integration of the second and third structure results into a novel time series detection technique. We use this integrated technique to identify multicategories EEG signals.

### 2.1. Sampling

**Figure 2:**
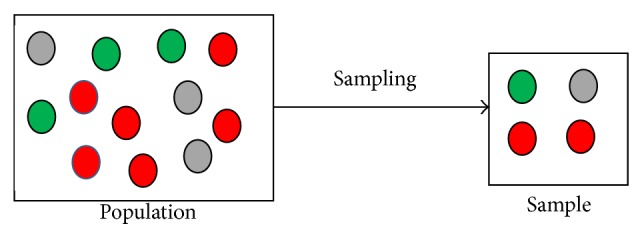
An example of a visual representation of the sampling process.

In statistics, sampling is a process of selection of a subset of individuals from a group of observations (called population) to represent the whole population.  Figure 2 illustrates how observations are selected in a sample from population. As shown in Figure 2, the population of size 12 consists of three colour observations such as red, green, and gray, where there are three elements of green colour, six elements of red colour, and three elements of gray colour. In the sample, two red, one green, and one gray colour elements are selected from the population through a random sampling process. Thus only four elements are selected in the sample that represents the whole population of size 12. In the proposed framework, before using sampling, we segment the EEG signals of each class into several groups based on a particular time period in order to have representative values of a specific time period.

The reason of segmentation is to properly account for possible stationarities assignal processing methods requiring stationarity of signals while EEG signals are nonstationary and aperiodic and the magnitudes of the signals are changed over time. In order to have representative values of a specific time period, the EEG signals of a class are divided into some mutually exclusive groups. As can be seen in Figure 1, this study partitions the EEG signals of each class into *k* nonoverlapping segments denoted by Seg_1_, Seg_2_,…, Seg_*k*_ considering a particular time period. Then, the representative observations are selected from each segment by the RS and OS technique, separately. Depending on the selection process, the algorithm consists of two types, provided below.

#### 2.1.1. Random Sampling (RS)

In this case, we determine the required sample size from each segment considering each segment as a population with a desired confidence interval and confidence level. The required sample size of the whole data of a class (called population) is determined by using (1) and (2) [13–16]:

(1) $$\begin{array}{c} \text{SS}=\frac{z^{2}\times p\times \left(1-p\right)}{e^{2}}, \end{array}$$

where SS means the sample size; *z* is the standard normal variate (*Z*-value) for the desired confidence level; *p* is the assumed proportion in the target population estimated to have a particular characteristic; and *e* is the margin of errors or the desired level of precision. If population is finite, the required sample size for each class is given by

(2) $$\begin{array}{c} n=\frac{\text{SS}}{1+\left(\text{SS}-1\right)/\text{Popu}}, \end{array}$$

where Popu means population size and *n* is the required sample size. After determining the sizes, we select the representative samples directly from the respective segments of each class. Then all of the selected samples from the segments of each class are combined together in a set (called RS set) from where representative characteristics are obtained as features discussed in Section 2.2.

#### 2.1.2. Optimum Allocation Sampling (OS)

In this scenario, we firstly determine the required sample size from the whole EEG signals with a desired confidence interval and confidence level. Then we determine the required sample from each segment using the optimum allocation (OS) scheme by (3) that considers the variability among the signals in each segment. A detailed description of the OS is available in [5, 14]:

(3) ni=Ni∑j=1psij2∑i=1kNi∑j=1psij2×mi=1,2,….,k; j=1,2,…,p,

where *n*(*i*) is the required sample size of the *i*th Seg; *N*_*i*_ is the data size of the *i*th Seg; *s*_*ij*_^2^ is the variance of the *j*th channel of the *i*th Seg; and *m* is the sample size of the EEG recording of a class obtained. Finally, we select the required sample from each segment based on the OS structure. Then all of the selected samples from the segments of each class are united in a set (named OS set) and representative characteristics are extracted from the OS set as discussed in Section 2.2.

### 2.2. Feature Extraction

Feature extraction aims at describing many data points into fewer parameters, which are termed “features” that represent important pattern of data distribution. The feature extraction process transforms the original signals into a feature vector. These features represent the behaviours of the EEG signals, which are particularly significant for recognition and diagnosing purposes. In this paper, the eleven statistical features from each segment of EEG channel data are extracted as the valuable parameters for the representation of the characteristics of the original EEG signals which are mean (*X*_Mean_), median (*X*_Me_), mode (*X*_Mo_), standard deviation (*X*_SD_), first quartile (*X*_Q1_), third quartile (*X*_Q3_), interquartile range (*X*_IQR_), skewness (*X*_*β*_1__), kurtoses (*X*_*β*_2__), minimum (*X*_*Min*⁡_), and maximum (*X*_*Max*⁡_). It is noted that these features are the most representative values to describe the original EEG signal in each segment. The feature set is denoted by {*X*_Mean_, *X*_Me_, *X*_Mo_, *X*_Q1_, *X*_Q3_, *X*_IQR_, *X*_SD_, *X*_*β*_1__, *X*_*β*_2__, *X*_*Min*⁡_, *X*_*Max*⁡_}. Out of above eleven features, *X*_*Min*⁡_, *X*_*Max*⁡_, *X*_Me_ (also called 2nd quartile), *X*_Q1_, and *X*_Q3_ are together called a five-number summary. A five-number summary is sufficient to represent a summary of a large dataset [17–19]. It is well known that a five-number summary from a database provides a clear representation about the characteristics of a dataset.

Again an EEG data can be symmetric or skewed. For a symmetric distribution, appropriate measures for measuring the centre and variability of the data are the mean and the standard deviation, respectively. For skewed distributions, the median and the interquartile range (IQR) are the appropriate measures for measuring the centre and spread of the data [17, 19]. Mode is the value in the dataset that occurs most often. The mode for a continuous probability distribution is defined as the peak of its histogram or density function.* Skewness* describes the shape of a distribution that characterizes the degree of asymmetry of a distribution around its mean [17, 19].* kurtosis* is a descriptor of the shape of a data distribution whether the data are peaked or flat relative. It quantifies whether the shape of the data distribution matches the normal distribution. For these reasons, we consider these eleven statistical features as the valuable parameters for representing the characteristics of the EEG signals and also brain activity as a whole. The accumulations of all obtained features from all segments of all classes are employed as the input for the three different classifiers.

### 2.3. Detection

In this work, this study employs three classifiers: *k*-nearest neighbours (*k*-NN), multinomial logistic regression with ridge estimators (MLR), and support vector machine (SVM) to evaluate the performance for the RS and OS feature set. The reason of choosing of these classifiers for this study is its simplicity and effectiveness in implementation. They is also very powerful and fastest learning algorithm that examines all its training input for classification in this area. The following sections provide a brief idea about the classification methods that are used in this research.

#### 2.3.1. *k*-Nearest Neighbours (*k*-NN)

The *k*-NN is a very intuitive method in which the classifier labels observations based on their similarity between observations in the training data. Among the various methods of supervised statistical pattern recognition, the *k*-NN rule achieves consistently high performance, without a priori assumptions about the distributions from which the training examples are drawn [20]. Given a query vector *x*_0_ and a set of *N* labelled instances {*x*_*i*_, *y*_*i*_}_1_^*N*^, the task of the classifier is to predict the class label of *x*_0_ on the predefined *P* classes. The *k*-NN classification algorithm tries to find the *k*-nearest neighbors of *x*_0_ and uses a majority vote to determine the class label of *x*_0_. Without prior knowledge, the *k*-NN classifier usually applies Euclidean distances as the distance metric [21]. An appropriate value should be selected for *k*, because the success of classification is very much dependent on this value. There are several methods to choose the* k*-value; one modest idea is to run the algorithm many times with different* k*-values (*k* = 1,2,…, 20) and choose the one with the best performance. A detailed discussion of this method is available in [22, 23].

#### 2.3.2. Multinomial Logistic Regression Classifier with a Ridge Estimator (MLR)

The MLR have become increasingly popular with the easy availability of appropriate computer routines. Ridge estimators are used in MLR to improve the parameter estimates and to diminish the error made by further prediction when maximum likelihood estimators (MLE) are nonunique and infinite to fit data. When the number of explanatory variables is relatively large and or when the explanatory variables are highly correlated, the estimates of parameters are unstable, which means they are not uniquely defined (some are infinite) and/or the maximum of log-likelihood is achieved at 0 [24, 25]. In this situation, ridge estimators are used to generate finiteness and uniqueness of MLE to overcome such problems. Let the response variable *Y* ∈ {1,2,…, *k*} have *k* possible values (categories). If there are *k* classes for *n* instances with *m* attributes (explanatory variables), the parameter matrix *B* to be calculated will be *m* × (*k* − 1). The probability for class *j* with the exception of the last class is

(4) $$\begin{array}{c} P_{j}\left(X_{i}\right)=\frac{\exp\left(X_{i}B_{j}\right)}{\left(\sum_{j=1}^{k}\exp\left(X_{i}B_{j}\right)+1\right)}. \end{array}$$

The last class has the probability

(5) $$\begin{array}{c} 1-\overset{k-1}{\underset{j=1}{\sum }}P_{j}\left(X_{i}\right)=\frac{1}{\sum_{J=1}^{K-1}\exp\left(X_{i}B_{j}\right)+1}. \end{array}$$

The (negative) multinomial log-likelihood is thus

(6) L=−∑i=1n∑j=1k−1Yij×In⁡PjXi+1−∑j=1k−1Yij1−∑j=1k−1PjXipppppppp×In⁡1−∑j=1k−1PjXi+ridge×B2.

In order to find the matrix *B* for which *L* is minimised, a Quasi-Newton Method is used to search for the optimized values of the *m* × (*k* − 1) variables [24]. Note that before we use the optimization procedure, we “squeeze” the matrix *B* into *m* × (*k* − 1) vector. A detailed description of the MLR can be found in [24, 25].

#### 2.3.3. Support Vector Machine (SVM)

The SVM is most popular machines learning tool that can classify data separated by nonlinear and linear boundaries, originated from Vapnik's statistical learning theory [26]. The main concepts of the SVM are to first transform input data into a higher dimensional space and then construct an optimal separating hyper plane (OSH) between the two classes in the transformed space [27, 28]. Those data vectors nearest to the constructed line in the transformed space are called the support vectors that contain valuable information regarding the (OSH). SVM is an approximate implementation of the “method of structural risk minimization” aiming to attend low probability of generalization error. In most real life problems (including our problem), the data are not linearly separable. In order to solve nonlinear problems, SVMs use a kernel function [27, 28], which allows better fitting of the hyperplane to more general datasets. In more recent times, SVMs have been extended to solve multiclass-classification problems. One frequently used method in practice is to use a set of pairwise classifiers, based on one-against-one decomposition [28]. The decision function for binary classification is as follows:

(7) $$\begin{array}{c} f\left(x\right)=\mathrm{sgn}\left(\overset{s}{\underset{i=1}{\sum }}y_{i}\alpha_{i}k\left(x_{i},x\right)+b\right);\;0<\alpha_{i}<C, \end{array}$$

where sgn is the signum function, *K*(*x*_*i*_, *x*) is kernel function, and *b* is the bias of the training samples. In this paper, radial basis function (RBF) kernel is considered as a choice for identifying different categories EEG signals because it was found to give the best classification performance. Here *C* is regularization parameter used to tune the trade-off between minimizing empirical risks (e.g., training error) and the complexity of the machine is always set to its default value; namely, *C* = *N*/∑_*i*=_^*N*^ *K*(*x*_*i*_, *x*), where *N* is the size of the training set.

In the multiclass classification, the SVMs work by using a collection of decision functions *f*_*kl*_, and here* kl* indicates each pair of classes selected from separated target classes. The class decision can be achieved by summing up the pairwise decision functions [28]

(8) $$\begin{array}{c} f_{k}\left(x\right)=\overset{n}{\underset{i=1}{\sum }}\mathrm{sgn}\left(f_{kl}\left(x\right)\right). \end{array}$$

Here *n* is the number of separated target classes. The algorithm proceeds as follows: assign a label to the class: arg max⁡*f*_*k*_(*x*), (*k* = 1,2,…, *n*). The pairwise classification converts the* n*-class classification problem into *n*(*n* − 1)/2 two-class problems which cover all pairs of classes. An overview of SVM pattern recognition techniques may be found in [26–28].

## 3. Data and Experimental Design

### 3.1. Data

**Figure 3:**
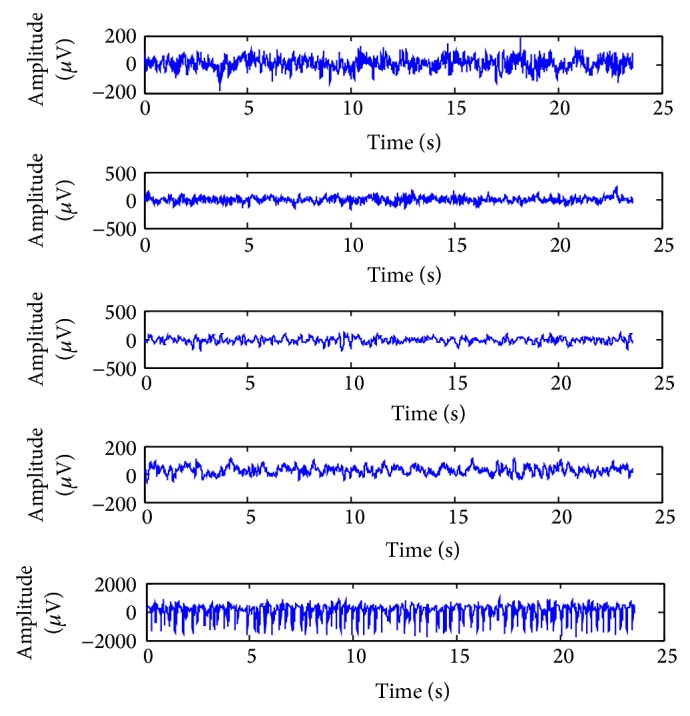
Exemplary EEG signals from each of the five sets. From top to bottom: class Z, class O, class N, class F, and class S.

We used the EEG time series database [29] which is publically available and is considered as a benchmark of testing classification techniques. The detailed descriptions of the dataset are discussed by Andrzejak et al. [30]. The whole database consists of five EEG datasets (Sets A–E), each containing 100 single channel EEG signals of 23.6 sec duration, composed for the study. Set A (denoted class Z) and Set B (denoted class O) consisted of segments taken from surface EEG recordings that were carried out on five healthy volunteers using a standardized electrode placement scheme. Volunteers were relaxed in an awake state with eyes open (class Z) and eyes closed (class O), respectively. Sets C, D, and E (denoted classes N, F, and S, resp.) originated from presurgical diagnosis. Segments in Set D (class F) were recorded from within the epileptogenic zone and those in Set C (class N) from the hippocampal formation of the opposite hemisphere of the brain. While Set C (class N) and Set D (class F) contained only activity measured during seizure free intervals, Set E (class S) only contained seizure activity. All EEG signals were recorded with the same 128-channel amplifier system, using an average common reference. After 12-bit analog-to-digital conversion, the data were written continuously onto the disk of a data acquisition computer system at a sampling rate of 173.61 Hz. Band-pass filter settings were 0.53–40 Hz (12 dB/oct.). In this work, five classes' (Z to S) classification problems, called multiclass classification, are performed from the above dataset in order to verify the performance of the proposed method. All the EEGs from the dataset are used and they are classified into five different classes: Z, O, N, F, and S, which can be denoted by Z-O-N-F-S. Exemplary EEGs of each of the five classes are depicted in Figure 3.

### 3.2. Training and Testing: Cross Validation

There are many choices of how to divide the data into training and test sets [31]. In order to reduce the bias of training and test data, we propose employing* k*-fold cross validation technique [31–34] considering *k* = 10 in this study. This technique is implemented to create the training set and testing set for evaluation. Generally, with* k*-fold cross validation, feature vector set is divided into *k* subsets of (approximately) equal size. The proposed classifiers are trained and tested *k* times, in which one of the subsets from training is left out each time and tested on the omitted subset. Each time, one of the subsets (folds) is used as a test set and the other *k* − 1 subsets (folds) are put together to form a training set. Then the average accuracy across all *k* trials is computed for consideration.

### 3.3. Select Optimum Values of the Parameters of the Classifiers

As mentioned before, this study uses three classification methods: *k*-NN, MLR, and SVM. The *k-*NN model has only one parameter *k* which refers to the number of nearest neighbors. By varying *k*, the model can be made more or less flexible. In this study, we select appropriate *k*-value in automatic process following *k* selection error log as there is no simple rule for selecting *k*. We consider the range of *k*-value in between 1 and 30 and pick an appropriate *k*-value that results in lowest error rate as the lowest error rate refers to the best model. In the experimental results, we obtain the lowest error rate for *k* = 1. In the MLR method, the parameters are obtained automatically through a ridge estimator discussed in Section 3.3. For the SVM, the RBF kernel function is employed as an optimal kernel function over different kernel functions that were tested. As there are no specific guidelines to set the values of the parameters for the MLR and the SVM classifiers, we consider the parameter values that have been used in WEKA default parameters settings.

### 3.4. Performance Evaluation of Classification Schemes

Criteria for evaluating the performance of a classifier are an important part in its design. In this study, we assess the performance of the proposed classifiers through most of the criteria that are usually used in biomedical research such as true positive rate (TPR) or sensitivity, false alarm rate (FAR) or false positive rate or 1 − specificity, precision, recall, *F*-measure, accuracy, kappa statistics, mean, receiver operating characteristic (ROC) curve area, and absolute error (MAE). These criteria allow estimating the behaviour of the classifiers on the extracted feature data. The evaluation measure most used in practice is accuracy rate which evaluates effectiveness of the classifier by its percentage of correct prediction [35–37]. The TPR (sensitivity) provides the fraction of positive cases that are classified as positive and it is also called recall [18, 31, 33, 38]. The FAR [5] is the percentage of false positives predicted as positive from negative class. The FAR usually refers to the expectancy of the false positive ratio. Precision (positive predictive value) is a measure which estimated the probability that a positive prediction is correct. *F*-measure is a combined measure for precision and recall calculated as 2∗Precision∗Recall/(Precision + Recall). Kappa is a chance-corrected measure of agreement between the classifications and the true classes [39]. It is calculated by taking the agreement expected by chance away from the observed agreement and dividing by the maximum possible agreement. The area under the ROC curve provides a measure of overall performance of the classifier. The ROC curve displays the plots of TPR (sensitivity) versus false positive rates [31]. Mean absolute error (MAE) is used to measure how close predictions are to the eventual outcomes.

## 4. Experimental Results and Discussions

To validate the effectiveness of the proposed approach, we examine this scheme on the epileptic EEG database. The analyses of the RS and OS application are presented in Section 4.1.  Section 4.2 reports the resultant classification performance of the proposed method. This section also provides a comparison between the proposed method and four well-known existing methods. All of the calculations are carried out in MATLAB (version 7.14, R2012a). We experimented three classification algorithms: *k*-NN, MLR with a ridge estimator, and SVM implemented in WEKA machine learning toolkit [40]. LIBSVM (version 3.2) [41] is used for the SVM classification in WEKA.

### 4.1. Analysis on the Application of RS and OS

According to our framework as shown in Figure 1, at first we segment each of the five classes into four parts (*k* = 4). As every channel of a class contains 4097 data points of 23.6 seconds, in each class, the sizes of the four segments, Seg_1_, Seg_2_, Seg_3_, and Seg_4_, are *N*_1_ = 1024, *N*_2_ = 1024, *N*_3_ = 1024, and *N*_4_ = 1025, respectively, and each segment contains the data for 5.9 sec. Then we select a sample (a representative subset of a segment) from each of the four segments in every class using the RS and OS technique, separately as discussed in Section 2.1. The calculated required sample sizes under the RS and OS technique are reported in Table 1. In the RS, the sample sizes for each segment are calculated by (2) whereas (3) is used to compute the sample sizes for each segment in the OS scheme. Using the calculated sample sizes displayed in Table 1, the samples are selected from the respective segments of that class. It is important to note that the sample selection procedure is repeated* twenty* times in both the RS and OS schemes to achieve most reliable and consistent results.

**Figure 4:**
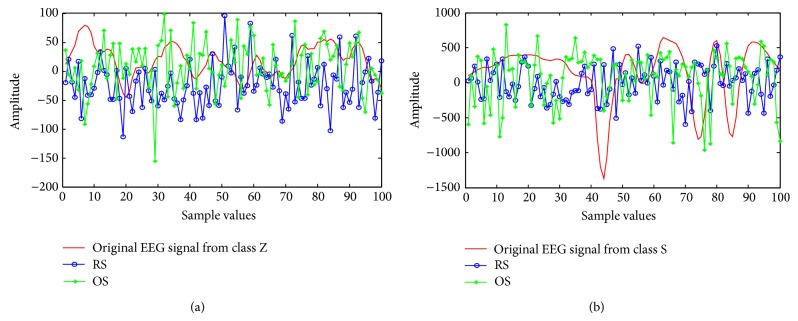
(a) Exemplary pattern of the RS and OS data with their respective original EEG signal from class Z (healthy subject with eye open). (b) Exemplary pattern of the RS and OS data with their respective original EEG signal class S (epileptic patient during seizure activity).

To illustrate exemplary pattern of the RS and OS sample, Figures 4(a) and 4(b) are presented for a segment of a class.  Figure 4(a) displays an exemplary pattern of the RS and OS with their respective original EEG signal from class Z (healthy subject with eye open). In Figure 4(a), we consider RS sample and OS sample of 100 observations and their respective original signal with same size from Seg_1_ of class Z to point out pattern of the RS and OS data with their original pattern. This figure reveals almost same pattern of the RS and OS sample with their respective original EEG signal.

Figure 4(b) presents an exemplary outline of the RS and OS data with an original signal from class S (epileptic patient during seizure activity). As in Figure 4(a), the RS sample and OS sample with 100 data points are considered from Seg_1_ of class S to show pattern of both samples with their respective original signal's pattern. As shown in Figure 4(b), the patterns of the RS and OS scheme are not very similar with their respective original signal.

After selection of the samples from each of the four segments of each and every class by the RS procedure, we combine all four samples of a class in a set called “RS” of that class and we perform similar process for the OS scheme and called it “OS” set of that class as shown in Figure 1. Then we extract eleven features separately from the “RS” set and the “OS” set of each class to represent the distribution pattern of that class. The reasons of considering the eleven features in this study are discussed in detail in Section 2.2. As each of the five classes consists of 100 single channel EEG signals, the size of feature vector for a class is 100 × 11 in both the RS and OS schemes. Thus the size of whole feature vector for all five classes is 500 × 11 in both sampling processes. After that, 10-fold cross validation process is employed to generate training set and testing set for performance evaluation of the proposed algorithm as described in Section 3.2. In each of the 10 iterations, the training set holds 450 × 11 data point while the testing set contains 50 × 11 data point. Here the training set is used to train the classifier and the testing set is used to evaluate the accuracy and the effectiveness of the classifiers for the detection of the multiclass EEG data.

**Figure 5:**
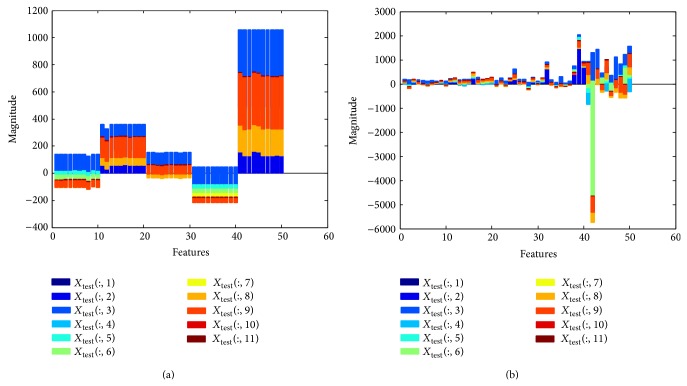
(a) Illustration of feature values for the RS scheme in a testing set. (b) Illustration of feature values for the OS scheme in a testing set.

To provide an idea about the feature sets, we present two diagrams: Figures 5(a) and 5(b) for the RS and OS scheme, respectively, illustrating features of a testing set (1st fold). As we know, the testing set contains five class features. In both Figures 5(a) and 5(b), these five classes features are plotted in *x*-axis indicating 1–10 for class Z, 11–20 for class O, 21–30 for class N, 31–40 for class F, and 41–50 for class S in both figures. We observe on these two diagrams that there are some quantitative differences between two sampling (RS and OS) features. In each classification system, the training set is fed into the three different classifiers as the input to train the classifier and the performances are assessed with the testing test.

### 4.2. Resultant Classification Performance

To explore the performance of the RS and OS features, we tested three machine leaning methods: *k*-NN, MLR with a ridge estimator, and SVM for detection of multicategory EEG signals. It is important to note that, due to the usage of sampling process, different samples may come in different occasions for both the RS and OS schemes. To overcome this bias and to achieve more reliable and consistent outcomes, the sampling procedure is repeated 20 times for both the schemes with all the classifiers used in this paper and then the average performance parameter values are reported.  Table 2 reports the detection performance for the *k*-NN classifier with the optimum *k*-value (*k* = 1) for both the RS and OS features, separately. This table provides different performance parameter values for each of the five classes in addition to the overall performance. In Table 2, it can be seen that there is a significant difference of performances of *k*-NN classifier between the RS and OS technique. As shown in Table 2, under the RS scheme, all of the performances indicators demonstrate perfect detection of five categories EEG signals by the *k*-NN classifier with zero FAR. In this case, all of the measurements of TPR, precision, recall, *F*-value, and accuracy for each and every class are 100% for the RS features. On the other hand, under the OS scheme, the performance of *k*-NN classifier is not satisfactory. In this case, the overall TPR, precision, recall, *F*-value, and accuracy for the OS features are 63.6%, 9.1%, 63.2%, 63.6%, 63.1%, and 63.6%, respectively, with varying FAR. The overall accuracy is increased 36.4% for the RS scheme compared to the OS scheme. The significant improvement is due to the fact of the use of the RS scheme, the statistical features that well represent the EEG signals compared to the OS scheme.

Tables 3 and 4 display the classification results of the MLR and SVM classifiers under both RS and OS approach. In both Tables 3 and 4, it is seen that the RS technique achieves better performances for each and every individual class with the MLR with very low FPR compared with the OS technique. As shown in Table 3, the overall accuracy is 99.80% for the RS based MLR approach, while it is 61.20% for the OS based MLR method. In this case, the performance is improved 38.6% for the RS scheme. We can also see in Table 4 that the RS technique achieves 99.40% of the overall accuracy for the SVM classifier whereas it is very low, 23.0%, for the OS scheme. As we can see, the RS approach consistently performs better for the *k*-NN, MLR, and SVM classifiers with very few FPR. On the other hand, the OS approach is continuously producing lower performances and higher FAR with these three classifiers. This may be due to that fact that, under the OS approach, the sampling procedures and the statistical features do not represent the whole EEG signals. According to the classification results as displayed in Tables 2–4, it is obvious that the RS process is the best way for achieving representative information from various categories EEG signals and the *k*-NN classifier is the top suited with the RS based features for detecting multicategories EEG signals.

**Figure 6:**
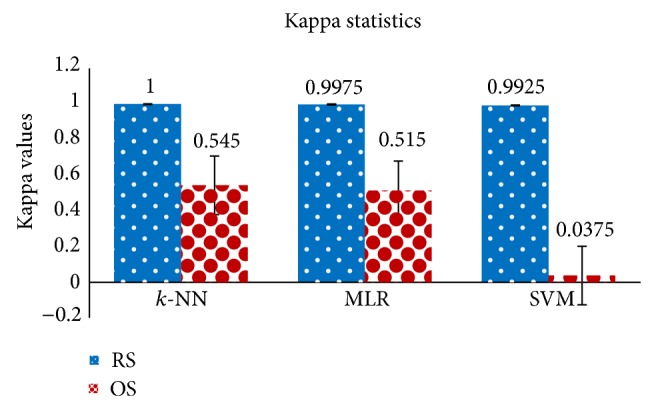
Kappa statistics values for the *k-*NN, MLR, and SVM classifier under the RS and OS scheme.

Figure 6 displays kappa statistics for the *k*-NN, MLR, and SVM classifier under the RS and OS scheme. In this research, kappa statistics test is used to evaluate the consistency of the three classifiers: *k*-NN, MLR, and SVN between the two processes, RS and OS scheme. The consistency is mild if kappa value is less than 0.2, fair if it lies between 0.21 and 0.40, moderate if it lies between 0.41 and 0.60, good if it is between 0.61 and 0.80, and excellent if it is greater than 0.81. As seen in Figure 6, kappa values are very high (close to 1) for the RS scheme compared to the OS scheme for all of the three classifiers. In this figure, error bars indicate the standard error and standard errors are very high in the OS scheme for each of the three classifiers that indicate inconsistency of the OS method. In Figure 6, it can be seen that the highest kappa value is obtained by the *k*-NN algorithm with the RS scheme. This clearly indicates that the performance of the RS scheme with the *k*-NN classifier is excellent for the detection of multicategory EEG signals.

**Figure 7:**
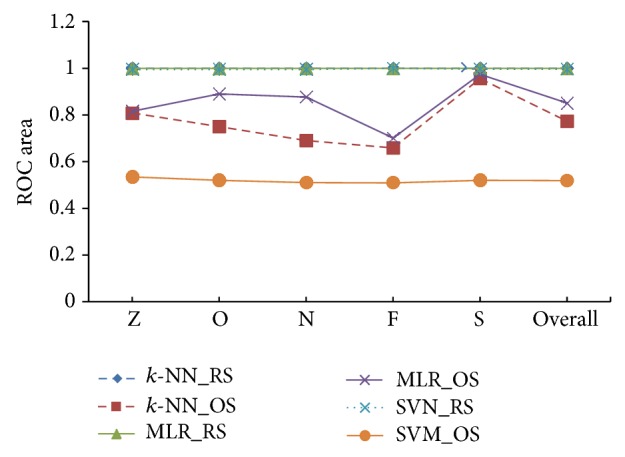
ROC area for the *k-*NN, MLR, and SVM classifier with the RS and OS scheme.

**Figure 8:**
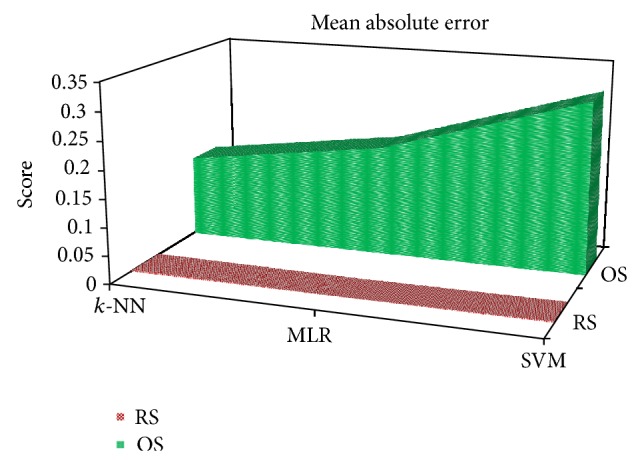
3D stacked area graph showing MAE for the *k*-NN, MLR, and SVM classifier under the RS and OS scheme.

Figure 7 presents ROC areas for the *k*-NN, MLR, and SVM classifiers with the RS and OS scheme, separately for each of five classes and their overall ROC area as well. The area of the ROC curve is used as an index for evaluating classifier performance (e.g., lager area indicates better performance of the classifier). As can be seen in Figure 7, each of the three classifiers produces higher ROC area close to 1 with the use of the RS scheme for each class while they yield lower area with the use of the OS scheme. This figure validates the reliability of the use of the RS scheme compared with the OS scheme to get representative sample point from the EEG data. The shape of the MAE for each of the three classifiers under the RS and OS scheme is illustrated in Figure 8. It is noted that the lower MAE score indicates the higher performance of the scheme. We can see that the score of MAE is very low for the RS approach for each of the three classifiers. On the other hand, the OS approach yields very high score of MAE for each of the classifiers. In this figure, we also observe that the lowest MAE is produced by the *k-*NN approach among the three classifiers for the RS scheme. Thus we can argue strongly that the statistical features obtained from RS scheme are perfect representation of EEG signals and the *k*-NN classifier is the best choice for multicategory EEG signals detection.

Plenty of promising research works have been devoted to the two-class classification problems providing very good outcomes dealing with the benchmark epileptic EEG data [18, 37, 42, 43] but a few studies in the literature [5, 6, 9–11] (discussed in Section 1) have been performed for the multiclass EEG signal classification. In order to further examine the efficiency of our proposed framework, we also provide a comparison of our proposed approach with five well-known reported algorithms.  Table 5 presents a comparative study between our proposed method and the five reference algorithms for the same benchmark epileptic EEG dataset. This table reports the detection performances of the five categories EEG signals in terms of class specific accuracy and overall accuracy. The highest classification performances among the five algorithms are highlighted in* bold* font in each method. From Table 5, it is clear that our proposed algorithm yields the perfect detection performances that are not achieved by any other methods in the literature. Thus, the RS scheme can be used as a perfect scheme for feature extractions while the *k*-NN can be considered as an optimum choice with it for the detection of multicategories EEG signals.

## 5. Concluding Remarks

Perfect detection of multicategory EEG signals is a complicated problem, requiring the analysis of large sets of EEG data. This study proposes a structure based on sampling and machine learning approach to detect multicategory EEG signals. The RS and OS scheme are employed to select representative samples from different segments of multicategory EEG signals. We experimented this methodology on benchmark epileptic EEG database. To examine the consistency of the structure, the sample selection procedure in both the RS and OS schemes with all the classifiers used in this paper is repeated for 20 times and the average performance parameter values are reported. The experimental results show that the features obtained from the RS well represent the multicategory EEG signals and achieve the consistent detection rates in terms of all possible detection parameters in all of the three classifiers used in this paper. The results also demonstrated that the *k*-NN classifier perfectly detects (100% for all performance indicator) the multicategory EEG signals under the RS technique. The results represent a proof concept of the successful detection of multicategory brain dynamics quantification through EEGs. Due to its perfect detection, the RS technique is strongly recommended for capturing the valuable information from the original EEG data which is best suited with the *k*-NN classifier. The proposed method may be applied for analysis and classification of other nonstationary biomedical signals.

## Figures and Tables

**Table 1:** Obtained sample sizes by the OS and RS technique from each segment of every class.

Different classes	Seg_1_⁡	Seg_2_⁡	Seg_3_⁡	Seg_4_⁡	Total
RS					
Class Z	965	965	965	966	3861
Class O	965	965	965	966	3861
Class N	965	965	965	966	3861
Class F	965	965	965	966	3861
Class S	965	965	965	966	3861
Total					**19305**

OS					
Class Z	797	822	837	832	3288
Class O	815	840	805	828	3288
Class N	839	841	780	828	3288
Class F	828	833	788	839	3288
Class S	833	844	815	796	3288
Total					**16440**

**Table 2:** Performances of the *k*-NN classifier on the RS and OS scheme.

	Performances for *k*-NN classifier											
Class	RS scheme						OS scheme					
	TPR	FAR	Precision	Recall	*F*-value	Acc	TPR	FAR	Precision	Recall	*F*-value	Acc
Z	100	0	100	100	100	100	72.0	10.5	63.2	72.0	67.3	72.0
O	100	0	100	100	100	100	63.0	13.0	54.8	63.0	58.6	63.0
N	100	0	100	100	100	100	49.0	11.0	52.7	49.0	50.8	49.0
F	100	0	100	100	100	100	41.0	9.3	52.6	41.0	46.1	41.0
S	100	0	100	100	100	100	93.0	1.8	93.0	93.0	93.0	93.0
Overall	100	0	100	100	100	100	63.6	9.1	63.2	63.6	63.1	63.6

**Table 3:** Performances of the MLR on the RS and OS scheme.

	Performances for MLR											
Class	RS scheme						OS scheme					
	TPR	FAR	Precision	Recall	*F*-value	Acc	TPR	FAR	Precision	Recall	*F*-value	Acc
Z	100	0	100	100	100	100	58.0	16.3	47.2	58.0	52.0	58.0
O	100	0.3	99.0	100	99.5	100	64.0	11.8	57.7	64.0	60.7	64.0
N	99.0	0	100	99.0	99.5	99.0	63.0	10.5	60.0	63.0	61.5	63.0
F	100	0	100	100	100	100	31.0	8.3	48.4	31.0	37.8	31.0
S	100	0	100	100	100	100	90.0	1.8	92.8	90.0	91.4	90.0
Overall	99.8	0.1	99.8	99.8	99.8	99.8	61.2	9.7	61.2	61.2	60.7	61.2

**Table 4:** Performances of the SVM with RBF kernel classifier on the RS and OS scheme.

	Performances for SVM with RBF kernel classifier											
Class	RS scheme						OS scheme					
	TPR	FAR	Precision	Recall	*F*-value	Acc	TPR	FAR	Precision	Recall	*F*-value	Acc
Z	99.0	0	100	99.0	99.5	99.0	7.0	0	100	7.0	13.1	7.0
O	99.0	0	100	99.0	99.5	99.0	4.0	0	100	4.0	7.7	4.0
N	99.0	0	100	99.0	99.5	99.0	2.0	0	100	2.0	3.9	2.0
F	100	0	100	100	100	100	2.0	0.3	66.7	2.0	3.9	2.0
S	100	0.8	97.1	100	98.5	100	100	96.0	20.7	100	34.2	100
Overall	99.4	0.2	99.4	99.4	99.4	99.4	23.0	19.3	77.5	23.0	12.6	23.0

**Table 5:** Comparison the results of our proposed approach with some reported research outcomes.

Methods	Description	Classification accuracy					Overall performance
		Class Z	Class O	Class N	Class F	Class S	
Proposed approach	RS + *k*-NN	**100.0**	**100.0**	**100.0**	**100.0**	**100**	**100.0**
Siuly and Li [5]	Optimum allocation + MLS-SVM	100.0	100.0	100.0	100.0	99.96	99.99
Shen et al. [6]	Wavelet-approximate entropy + SVM	100.0	100.0	99.87	100.0	100.0	99.97
Übeyli [9]	Wavelet coefficients and power spectral density (PSD) values + SVM	99.25	99.13	99.25	99.38	99.00	99.20
Übeyli [10]	PSD values + SVM	99.38	99.25	99.13	99.50	99.25	99.30
Guler and Ubeyli [11]	Wavelet coefficients and Lyapunov exponents + SVM	99.25	99.38	99.25	99.38	99.13	99.28
